# Structural Insights into a Unique *Legionella pneumophila* Effector LidA Recognizing Both GDP and GTP Bound Rab1 in Their Active State

**DOI:** 10.1371/journal.ppat.1002528

**Published:** 2012-03-01

**Authors:** Wei Cheng, Kun Yin, Defen Lu, Bingqing Li, Deyu Zhu, Yuzhen Chen, Hao Zhang, Sujuan Xu, Jijie Chai, Lichuan Gu

**Affiliations:** 1 State Key Laboratory of Microbial Technology, Shandong University, Jinan, Shandong, China; 2 Key Laboratory for Protein Sciences of Ministry of Education, School of Biological Sciences, Tsinghua University, Beijing, China; 3 Shandong Institute of Parasitical Diseases, Shandong Academy of Medical Sciences, Jining, Shandong, China; 4 Shandong Center for Disease Control and Prevention, Jinan, Shandong, China; Tufts University School of Medicine, United States of America

## Abstract

The intracellular pathogen *Legionella pneumophila* hijacks the endoplasmic reticulum (ER)-derived vesicles to create an organelle designated *Legionella*-containing vacuole (LCV) required for bacterial replication. Maturation of the LCV involved acquisition of Rab1, which is mediated by the bacterial effector protein SidM/DrrA. SidM/DrrA is a bifunctional enzyme having the activity of both Rab1-specific GDP dissociation inhibitor (GDI) displacement factor (GDF) and guanine nucleotide exchange factor (GEF). LidA, another Rab1-interacting bacterial effector protein, was reported to promote SidM/DrrA-mediated recruitment of Rab1 to the LCV as well. Here we report the crystal structures of LidA complexes with GDP- and GTP-bound Rab1 respectively. Structural comparison revealed that GDP-Rab1 bound by LidA exhibits an active and nearly identical conformation with that of GTP-Rab1, suggesting that LidA can disrupt the switch function of Rab1 and render it persistently active. As with GTP, LidA maintains GDP-Rab1 in the active conformation through interaction with its two conserved switch regions. Consistent with the structural observations, biochemical assays showed that LidA binds to GDP- and GTP-Rab1 equally well with an affinity approximately 7.5 nM. We propose that the tight interaction with Rab1 allows LidA to facilitate SidM/DrrA-catalyzed release of Rab1 from GDIs. Taken together, our results support a unique mechanism by which a bacterial effector protein regulates Rab1 recycling.

## Author Summary

*Legionella pneumophila* delivers 275 validated substrates into the host cytosol by its Dot/Icm type IV secretion system. Several substrates including SidM/DrrA and LidA directly interact with the host Rab GTPases and interfere with the vesicle secretion pathway. SidM/DrrA is necessary for Rab1 recruitment, function as a Rab1 specific GDI displacement factor and guanine nucleotide exchange factor. LidA has the auxiliary activity for Rab1 recruitment, whereas it is more important for the formation of the replication vacuole compared with SidM/DrrA. LidA is predicted to be the first substrate secreted by the Dot/Icm system and is critical for maintaining the integrity of the bacterial cell. Moreover, it expresses throughout the intracellular growth phase, localizes to early secretory compartments, and interacts with several members of Rab family. Here we present the crystal structures of LidA coiled-coil domain in complex with two different states of Rab1, GDP- and GTP-bound. The GDP-bound Rab1 in the complex surprisingly has the same conformation with the GTP-bound Rab1, revealing that LidA can retain Rab1 persistently in its active state. Our structures add a new insight into the regulation of the host Rab1 membrane cycle by pathogen-secreted coiled-coil effector.

## Introduction

Rab GTPases play a crucial role in vesicular trafficking through shuttling between cytosol and membranes, a process that is controlled by several regulatory proteins [1]–[4]. GDP dissociation inhibitors (GDIs) preferentially interact with and deliver GDP-bound Rabs to their target membranes, where dissociation of the GDI-Rab complexes is catalyzed by GDI displacement factors (GDFs) [5]–[8]. Prenylation at the C-termini of Rab proteins is essential for their membrane association [9]–[11]. The membrane-localized Rabs are subsequently activated by specific guanine nucleotide exchange factors (GEFs) via promoting their exchange of GDP for GTP [12]–[15]. The activated Rabs then bind their cognate effectors, triggering signaling for vesicle formation [16], [17], vesicle transport [18]–[23], vesicle tethering and fusion of vesicles with target membranes [17], [24]–[27]. GTPase activating proteins (GAPs) catalyze hydrolysis of GTP in the activated Rabs and return them to the GDP-bound inactive form that is sensitive to membrane retrieval by GDIs, thus replenishing the cytoplasmic pool of Rab proteins [4], [14], [15].

The intracellular bacterial pathogen *Legionella pneumophila* (*L. pneumophila*) is the causative agent of pneumonia Legionnaires disease [8], [28]. Following invasion of host cells, *L. pneumophila* resides in the LCV [29] that escapes endolysosomal destruction [30]. The bacterial effector proteins, delivered by the type IV secretion system (T4SS) of *L. pneumophila* into the cytosol of host cells [31], actively remodel the LCV to establish an intracellular niche indispensable to bacterial pathogenesis [32]. For example, the early secretory vesicles from ER can be hijacked to the LCV, converting it into an ER-derived organelle that supports bacterial replication [33]–[36]. Rab1, required for vesicle trafficking between ER and the Golgi complex [18], [37], [38], is one of the host proteins recruited to the LCV shortly after uptake of *L. pneumophila* [39]–[41]. Rab1 recruitment subsequently promotes transport and fusion of ER-derived vesicles with the LCV, thus playing an essential role in the biogenesis of this organelle [42], [43]. The effector protein SidM/DrrA, by acting as a Rab1-sepcific GEF and GDF, is required for LCV recruitment of Rab1 [44], [45]. Further, the AMPylation activity of SidM/DrrA modified Rab1 by covalently adding adenosine monophosphate (AMP) to avoid the GAP recognition [46]. SidD, functions as the Rab1 deAMPylase, generating de-AMPylated Rab1 accessible for inactivation by LepB [43], [47]. Another translocated effector protein LidA is also involved in the recruitment of early secretory vesicles to the LCV [48], [49]. LidA was found to interact with Rab1 as well, regardless of nucleotide binding states, and promote SidM/DrrA activity of transporting Rab1 to the LCV [39], [45]. Unlike SidM/DrrA mutants, however, *L. pneumophila* mutants lacking LidA displayed a temporal delay but not a loss of Rab1 recruitment to the LCV [39]. Intriguingly, while indispensable to the recruitment of wild type Rab1, SidM/DrrA is not required to accumulate Rab1 mutant (D44N) that loses interaction with GDIs but not with LidA about the LCV [45]. These results suggest that GDIs play a negative role in Rab1 recruitment by the LCV during *L. pneumophila* infection. Recruitment of this Rab1 mutant, however, is dependent on LidA, suggesting that interaction with Rab1 is important for the role of LidA in delivering Rab1 to the LCV. Currently the mechanisms of how LidA cooperates with SidM/DrrA for Rab1 recruitment are not well understood.

Here, we present the crystal structures of LidA (residues 224-559) in complex with a GDP-bound Rab1 mutant (S25N; residues 1-176) [50], [51] and LidA (residues 188-449) in complex with GTP-bound Rab1 (residues 1-176). Unexpectedly, the structures showed that GDP-Rab1(S25N), a “constitutively” inactive mutant, adopted an active conformation when bound by LidA. In agreement with the structural observation, biochemical assays demonstrated that GDP-Rab1(S25N) and GTP-Rab1(Q70L) [52] exhibited a similar and exceptionally high binding affinity for LidA. Coupled with previous observations, data presented in current study support a unique mechanism by which LidA interferes with the host secretory vesicular trafficking.

## Results

### LidA binds both GDP and GTP bound Rab1 with a similar affinity

**Figure 1:**
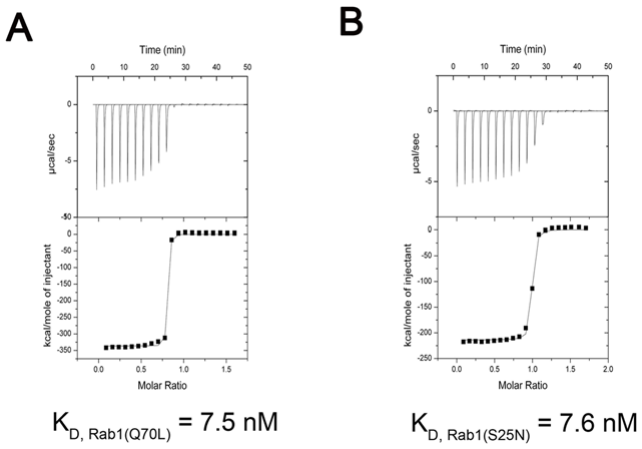
Measurement of binding affinity between LidA and GTP-bound or GDP-bound Rab1 by ITC. (A) Raw ITC data. Top panel: twenty injections of GTP-bound Rab1(Q70L) solutions were titrated into LidA(188-580) solution in ITC cell. The area of each injection peak corresponds to the total heat released for that injection. Bottom panel: the binding isotherm for GTP-bound Rab1(Q70L) and LidA(188-580) interaction, the integrated heat is plotted against the stoichiometry of 1∶1, data fitting revealed a binding affinity of 7.5 nM. (B) Top panel: twenty injections of GDP-bound Rab1(S25N) solutions were titrated into LidA(188-580) solution in ITC cell. The area of each injection peak corresponds to the total heat released for that injection. Bottom panel: the binding isotherm for GDP-bound Rab1(S25N) and LidA(188-580) interaction, the integrated heat is plotted against the stoichiometry of 1∶1, data fitting revealed a binding affinity of 7.6 nM. Both titrations were performed in the absence of added Mg^2+^ and GDP/GTP.

The Rab1-interacting domain of LidA used in our study is similar to the one (residues 191-600) necessary and sufficient to disrupt the secretory pathway when overexpressed in COS1 cells [49]. Consistently, *In vitro* data shows truncation of 188 residues from the N-terminal side or 155 residues from the C-terminal side of LidA generated no effect on the formation of such a complex (Figure S1). To further validate the interaction between LidA and Rab1, we measured their binding affinity using isothermal titration calorimetry (ITC) technique. The ITC results showed that the LidA fragment 188-580 containing the predicted coiled-coil domain interacted with the constitutively active mutant Rab1(Q70L) with a dissociation constant of 7.5 nM (Figure 1A). Surprisingly, the same protein also bound nearly equally well to the constitutively GDP-bound mutant of Rab1(S25N), with a dissociation constant of 7.6 nM (Figure 1B). A preference of LidA for the GTP-Rab1 could result from a lower abundance of GDP-Rab1 in cells.

### Overall structures of LidA and its complex with Rab1

To reveal the molecular basis for the LidA-Rab1 interaction, we first solved the crystal structure of LidA(224-559) in complex with a constitutively GDP-bound mutant Rab1(S25N; 1-176) at 1.73 Å using molecular replacement (Table S1 in supporting information).

**Figure 2:**
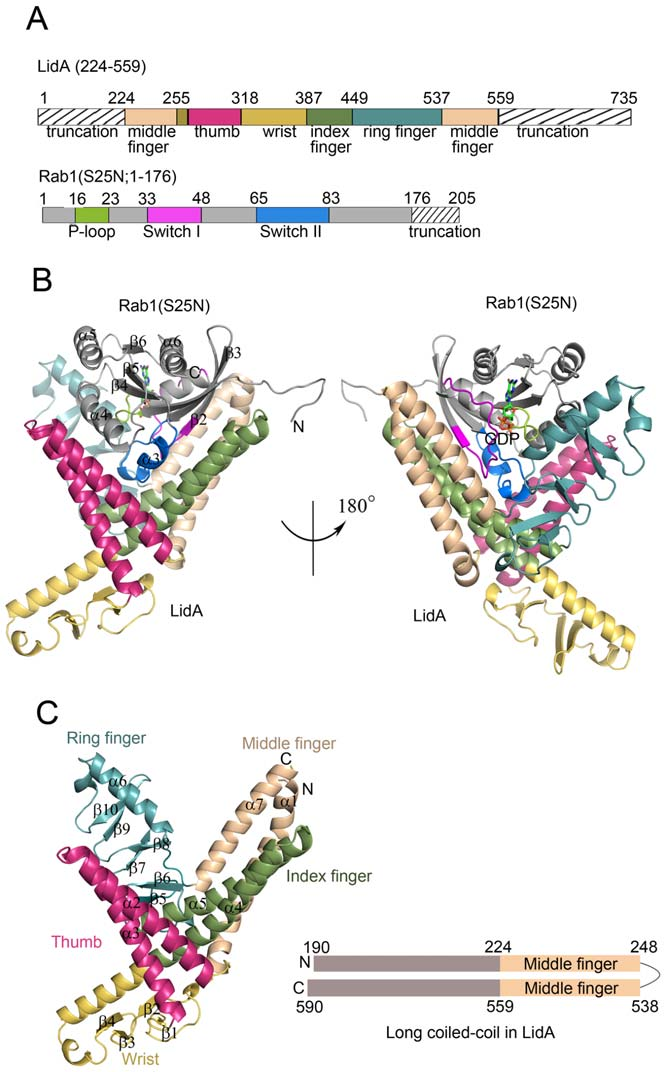
Overall structures of LidA and its complex with GDP-bound Rab1. (A) Schematic representation of LidA fingers and Rab1 function regions, sequences not included in the crystallized proteins are marked with hatched lines and labeled as truncation. (B) Cartoon representation of the overall structure of LidA(224-559)-Rab1(S25N; 1-176) complex. Rab1(S25N) is shown in gray, LidA is colored by fingers as shown in schematic representation, GDP is depicted in sticks. (C) Cartoon representation of the LidA fingers (left) and schematic representation of LidA terminal long coiled-coil domain (right), wheat color represents coiled-coil region in crystal structure, gray color represents extend α-helix predicted by secondary structure analysis. N, N terminus; C, C terminus.

The overall LidA-Rab1 complex adopts a compact and globular structure (Figures 2A,B). The interaction between LidA and Rab1 resulted in a 1∶1 stoichiometric complex and buried 41% (4031 Å^2^/9845 Å^2^) of the Rab1. The exceptionally large buried surface generated by LidA-Rab1 interaction is consistent with their strong binding affinity. In the structure, Rab1 is held in a large and pronounced groove made by the four “fingers” with an extensive, though not complete, charge and surface complementarities. Contacts between LidA and Rab1 are established through a combination of hydrogen bonds and hydrophobic interactions involving the switch I, switch II and the interswitch region of Rab1 (Figure 2B).

LidA (residues 224-559) is composed of seven α-helices and ten β-strands, which arrange into a structure resembling a hand with the baby finger buried in the palm and the remaining four straightened (Figure 2C). Six of the seven α-helices form three extended two-stranded α-helical coiled-coils. This structural observation supports a previous prediction that LidA is a coiled-coil rich structure [48], [49]. Two of them, made by α4/α5 and α1/α7, are the “index finger” and the “middle finger”, respectively. Interaction between them is primarily mediated by packing of α1 against α5. The “thumb” stems from a two-helix coiled-coil formed by α2 and α3 helices which interact with the base of “index finger” through packing against α4. The isolated helix α6 and its following loop make extensive hydrophobic contacts with three anti-parallel β-sheets (β5/β6, β7/β8 and β9/β10), resulting in formation of the “ring finger”. The N-terminal side of α4, which is not involved in the formation of coiled-coil structures, tightly contacts the two anti-parallel β-sheets (β1/β2 and β3/β4) located between α3 and α4, thus making the “wrist”. Interestingly, the α1 and α7 stay closely together and form the middle finger. The terminal parallel helix of LidA extends to the outside of interacting region, forms an antiparallel long coiled-coil (Figure 2C). So we suppose the N- and C-terminal domains of LidA may stay spatially close cooperate with each other to perform specific function.

### Interaction interfaces between LidA and Rab1

**Figure 3:**
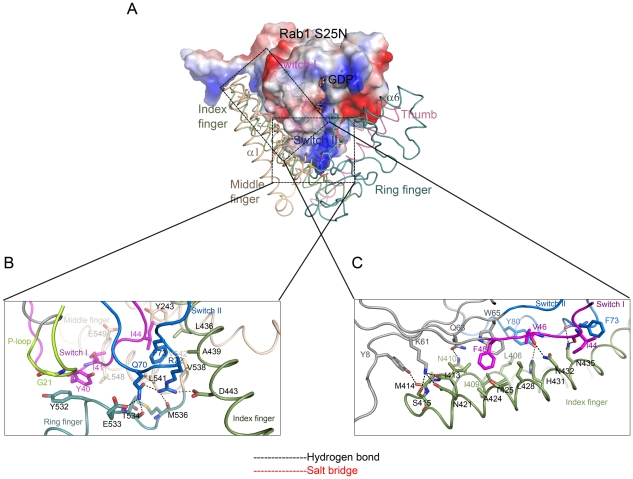
Interaction interfaces between LidA and Rab1. (A) Rab1(S25N) is shown with electrostatic surface potentials. Blue and red represent the positive and negative charge potential, respectively. The extensive interactions between Rab1(S25N) switch regions and LidA index finger (smude), middle finger (wheat) and ring finger (light teal) are shown. This Figure is in the same orientation as the right panel of Figure 2B. (B) The detailed interactions between Rab1 switch I (magentas), switch II (marine), P-loop (limon) and LidA middle finger and ring finger. (C) The detailed interactions between Rab1 switch II and LidA index finger. The interacting residues of Rab1 and LidA are shown in sticks, residues labels are color-coded as corresponding cartoon chains, respectively. Hydrogen bonds are indicated by black dashed lines. Salt bridge is indicated by red dashed lines.

In the complex, switch I (residues 33-48) of Rab1 is mainly sandwiched between the “middle finger” and the “ring finger” by making contacts with α1, α7 and α6. It also contacts the “index finger” α5 (Figures 3A,B). Close packing of Ile44^Rab1^ from switch I and Phe73^Rab1^ from switch II (residues 65-83) against Leu541^LidA^, Val542^LidA^, Val538^LidA^, Leu436^LidA^, Ala439^LidA^, Y243^LidA^ and the aliphatic portion of Asn432^LidA^ (from α5) appears to dominate the interactions around this interface. Additional van der Waals interactions result from contacts of Tyr532^LidA^ with the alpha carbon atom of Gly21^Rab1^ and Tyr40^Rab1^, and Ile41^Rab1^ with Leu548^LidA^ and Glu549^LidA^ in LidA. Hydrogen bonds further strengthen this interface. In particular, in addition to forming a salt bridge with Asp443^LidA^, Arg72^Rab1^ from the switch II region makes a hydrogen bonds with the backbone carbonyl of Met536^LidA^. Through an intramolecular hydrogen bond, Arg72^Rab1^ is stabilized by the catalytically important residue Gln70^Rab1^, which in turn mediates a bifurcated polar interaction with the carbonyl oxygen of Thr534^LidA^ and Glu533^LidA^ from LidA.

The C-terminal portion of the switch II, together with the region defined by β2, β3 and β4, makes extensive contacts with the base of “index finger” (Figures 3A,C). Center to this interface are the van der Waals interactions made by Phe48^Rab1^ and Trp65^Rab1^ from Rab1 with a cluster of surrounding hydrophobic residues of LidA. At periphery, a number of hydrogen bonds flank the hydrophobic interaction center. Among these, Lys61^Rab1^ from Rab1 forms three hydrogen bonds with Asn421^LidA^ and the carbonyl oxygen atoms of Ile413^LidA^ and Ser415^LidA^. Other hydrogen bonds include those formed by Tyr8^Rab1^ with the carbonyl oxygen atom of Met414^LidA^, Gln63^Rab1^ with Asn410^LidA^, the carbonyl oxygen atoms of Val46^Rab1^, ILE44^Rab1^ and Phe73^Rab1^ with Asn432^LidA^ and Asn435^LidA^, respectively. In addition to hydrogen bonding with His431^LidA^, Tyr80^Rab1^ also engages hydrophobic contact with Leu406^LidA^.

### The GDP-bound Rab1(S25N) is held in the active conformation upon LidA binding

**Figure 4:**
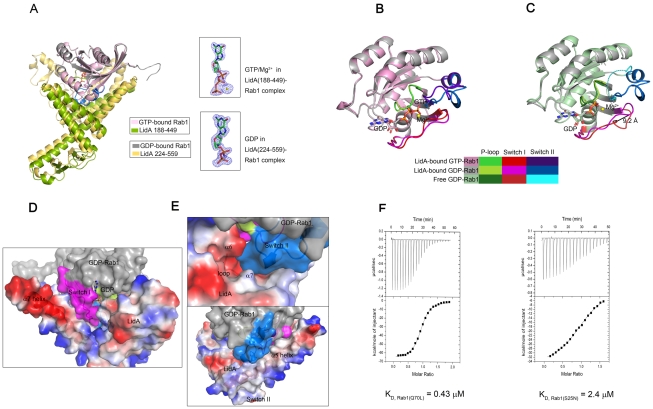
The complexed GDP-bound Rab1 (S25N) is held in the active conformation. (A) Superimposition of GDP-bound Rab1(S25N)-LidA complex and GTP-bound Rab1-LidA complex (left). The Fo-Fc electron density of GDP and GTP/Mg^2+^ in the two complexes is shown (right), respectively. The Figure demonstrates the quality of the electron density (blue mesh). Both of the electron density are depicted at 3.0 σ. (B) Superimposition of LidA-bound GDP-Rab1(S25N) and LidA-bound GTP-Rab1(WT). (C) Superimposition of LidA-bound GDP-Rab1(S25N) and free GDP-Rab1 (PDB ID code 2FOL). The largest displacement of 9.2 Å at the Cα atom of Ile in Switch I is shown. The disordered broken switch II in 2FOL is shown by blue dashed lines. The switch regions and P-loops are highlighted in different colors indicated in the color-box to contrast their conformational changes. (D and E) The C-terminal region of LidA contributes to maintain the GDP-bound Rab1(S25N) in active conformation. LidA(418-559) is shown with electrostatic surface potentials. (D) GDP-Rab1 switch I region is stabilized through the interaction with LidA α7 helix. (E) Switch II region is stabilized both by polar interaction with the loop linking LidA α6 and α7 and hydrophobic contacts with LidA α5 helix; Blue and red represent the positive and negative charge potential, white represent the hydrophobic contacts. Switch regions are color-coded as before. (F) Quantitative analysis of two ITC runs: titration of GTP-bound Rab1(Q70L) (left) and GDP-bound Rab1(S25N) (right) into LidA(188-449), respectively. Both titrations were performed in the absence of added Mg^2+^ and GDP/GTP. The *K_D_* value is 0.43 µM for GTP-bound Rab1(Q70L) and 2.4 µM for GDP-bound Rab1(S25N), respectively.

Our biochemical assay (Figures 1A,B) indicates that LidA binding is independent of the Rab1 nucleotide-binding states. To understand the underlying molecular mechanisms, we solved the crystal structure of LidA (residues 188-449) in complex with the Rab1(WT; residues 1-191) at 2.2 Å. The structure of the complex is highly similar to that of LidA(224-559)-Rab1(S25N) with an r.m.s.d of 0.678 Å for Rab1; 1.496 Å for LidA, respectively (Figure 4A). As anticipated, GTP is well defined in the crystal structure (Figure 4A) and its interactions with Rab1 are conserved in other active Rabs (Figure S2) [53]–[55]. Surprisingly, structural comparison revealed that the conformation of LidA-bound Rab1(S25N) is essentially identical with that of LidA-bound GTP-Rab1 (Figure 4B), indicating that Rab1(S25N) is in the active conformation following LidA binding. While the switch II region in the free GDP-Rab1 is completely disordered (Figure 4C), it is well defined when bound by LidA. As with GTP, LidA binding induces striking structural remodeling around the switch I region of Rab1(S25N), which swings from the edge of β-sheet to the nucleotide-binding site, with the largest displacement of 9.2 Å at the Cα atom of Ile. Relocation of switch I results in formation of an extended β-strand β2 at its N-terminal side and hydrophobic packing against switch II (Figure 4C).

Our structural analyses suggest that LidA utilizes a similar mechanism to GTP for maintaining Rab1 in the active conformation. The hydrogen bonding interactions between the γ phosphate group of GTP with Thr43 and Gly69, from switch I and switch II of Rab1 respectively, are highly conserved among Rab proteins and important for stabilizing their active state (Figure S2). Despite loss of the γ phosphate group-mediated interactions, the switch I region in Rab1(S25N) is stabilized through its interaction with α7 of LidA (Figure 4D). Stabilization of the switch II region in Rab1(S25N) is via its polar interaction with the loop linking α6 and α7 as well as hydrophobic contacts with α5 of LidA (Figure 4E). Deletion of the switch-stabilizing structural elements would be expected to disfavor LidA interaction with the GDP-bound Rab1 as compared to the GTP-bound Rab1. We therefore quantified LidA (residues 188-449) interaction with Rab1(S25N) and Rab1(Q70L) using an ITC assay. In support of the structure-based prediction, the assay showed that the LidA fragment exhibited a higher binding affinity (0.43 µM) to the GTP-bound Rab1 than to the GDP-bound Rab1 (2.4 µM) (Figure 4F).

### Interaction of Rab1-LidA shares similar features with that of Rab4-Rabenosyn-5

**Figure 5:**
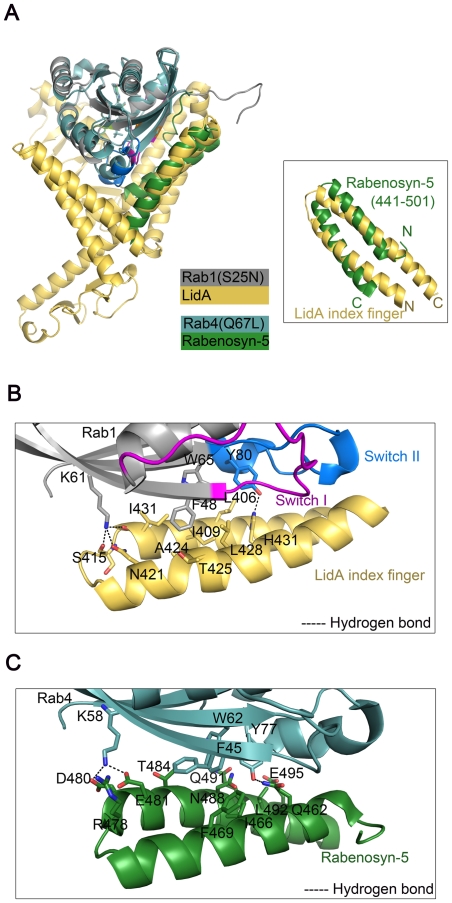
Interaction of Rab1-LidA shares similar features with that of Rab4-Rabenosyn-5. (A) Superimposition of Rab1(S25N)-LidA(224-559) complex with Rab4-Rabenosyn-5 complex (PDB ID code 1Z0K). The superimposition of the similar anti-parallel coiled-coil regions is shown. Rab1(S25N) is in gray and LidA is in yellow orange; Rab4 is in light blue and Rabenosyn-5 is in green. (B and C) Similar interfaces of the two complexes. (B) represents the detailed interactions between Rab1(S25N) and LidA. (C) represents the detailed interactions between Rab4 and Rabenosyn-5. The residues involved in interaction are shown as stick representations and denoted by black labels.

Interaction of the anti-parallel coiled-coil formed by the helices α4 and α5 with the switch and interswitch regions of Rab1 is reminiscent of the structure of Rab4-Rabenosyn-5 complex [54]. A superposition of the overall structures of these two complexes revealed significant structural similarity (Figure 5A). Some subtle structural differences surrounding the switch I and II are consistent with the notion that the switch regions adopt specific conformations in the GTP-binding form of Rab proteins [55]. Rabenosyn-5 forms a similar anti-parallel coiled-coil to LidA and binds the switch and interswitch regions of Rab4. Interestingly, although these two peptides have reverse orientations, they contact a significant subset of the highly conserved resides between Rab1 and Rab4, though the detailed molecular interactions differ. For example, in both complexes the highly conserved residues Phe48^Rab1^ (Phe45^Rab4^) and Trp65^Rab1^ (Trp62^Rab4^) are buried in a cluster of hydrophobic residues despite their different side chain rotamer conformations, whereas Lys61^Rab1^ (Lys58^Rab4^) and Tyr80^Rab1^ (Tyr77^Rab4^) are involved in polar interactions (Figures 5B,C).

### LidA interacts with multiple Rabs

**Figure 6:**
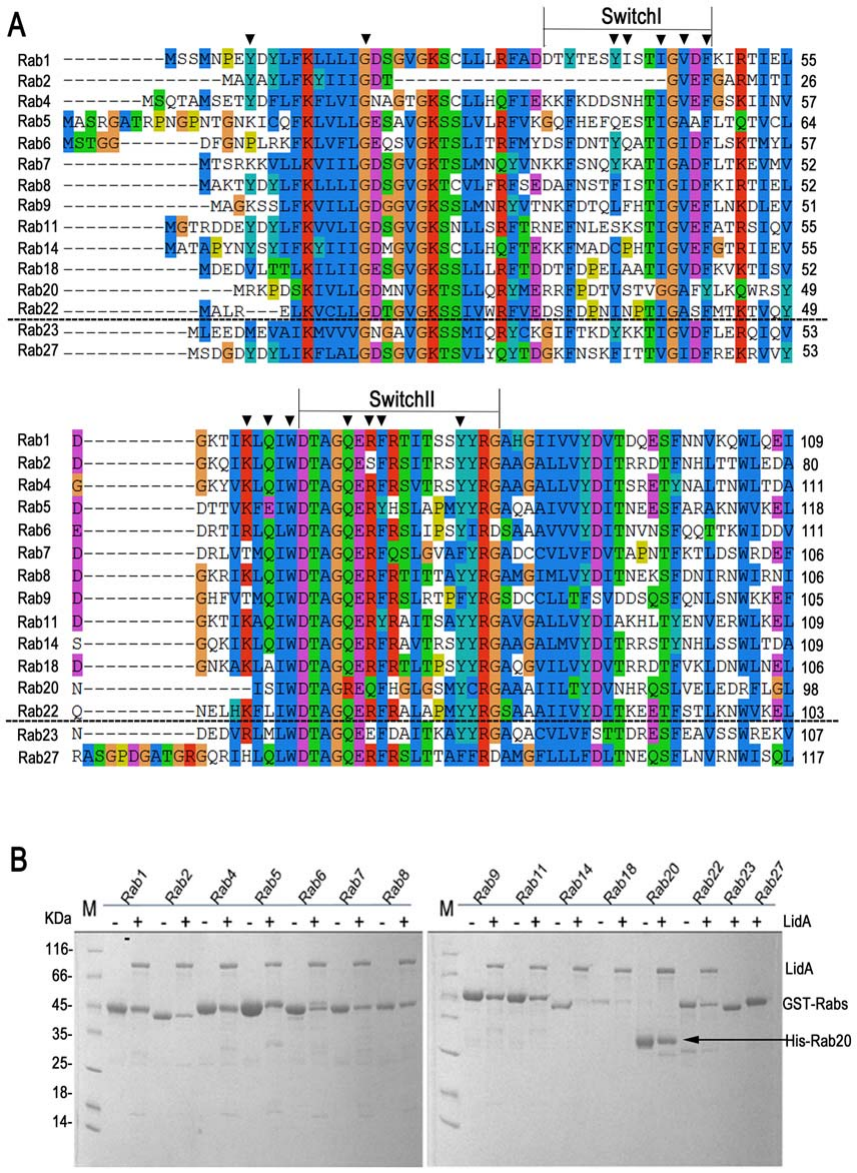
13 kinds of Rab GTPase family members could be recognized by LidA *in vitro*. (A) Sequence alignment of fifteen different Rabs. The LidA-interacting residues in Rab1 are depicted by triangles. The two sequences underneath the black dashed lines correspond to the members unbound to LidA. (B) The GST-pull down of LidA(FL) by beads-immobilized 15 kinds of GST-Rab family members, Rab20 was His-tagged for exceptional.

**Figure 7:**
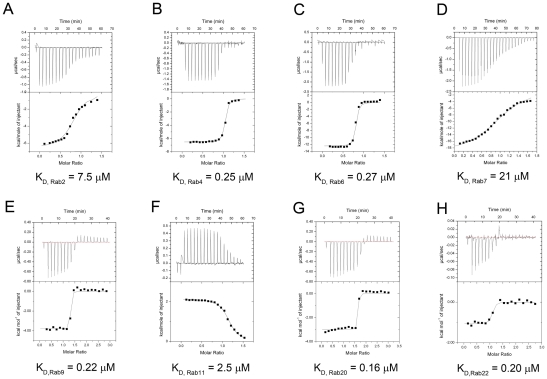
Measurement of binding affinity between LidA and 9 kinds of Rabs by ITC. (A–G) Raw ITC data. Top panel: twenty injections of Rab2 (A), Rab4 (B), Rab6 (C), Rab7 (D), Rab9 (E), Rab11 (F), Rab20 (G) solutions were titrated into LidA(188-580) solution in ITC cell. The area of each injection peak corresponds to the total heat released for that injection. Bottom panel: the binding isotherm for these Rabs and LidA(188-580) interaction, the integrated heat is plotted against the stoichiometry of 1∶1, data fitting revealed a binding affinity as shown. (H) Raw ITC data. Top panel: twenty injections of Rab22 (H) solutions were titrated into LidA(FL) solution ITC cell, the experiment conditions was exactly the same as the top one unless the LidA is full-length. The *K_D_* values of these Rabs and LidA are shown.

In addition to Rab1, LidA has been shown to be able to interact with other Rab member proteins such as Rab6a and Rab8b [39]. Indeed, many LidA-interacting residues in Rab1 are highly conserved in these two Rabs as well as other Rab members (Figure 6A) suggesting that LidA may have promiscuity of binding Rab proteins. To further experimentally test this, we purified 15 GST-fused Rab family members (His-fused Rab20 is exceptional) and tested their interaction with LidA using pull down assay. As shown in Figure 6B, thirteen out of the fifteen Rabs tested interacted with LidA. The binding affinities of LidA for other Rabs, including Rab2, Rab4, Rab6, Rab7, Rab9, Rab11, Rab20 and Rab22 have been quantified by ITC. All give *K_D_* values in the micromolar range. Compared with them Rab1 gives the strongest binding affinity (The *K_D_* is in nanomolar range). Thus Rab1 should be the preferred substrate of LidA compared with other Rabs (Figure 7). Thus, although previous LCV analysis identified interacting Rab GTPases such as Rab1, Rab7, Rab8, and Rab14 as novel LCV components [56], the biological relevance of the interactions await further investigation. To make clear how LidA choose substrate from so many Rabs, we attempted to disrupt the interactions of Rab1 and LidA by making single or double mutations of Rab1 and LidA along the surface of the interactions. However, none of the mutants we have tested by the pull-down assay lose their interaction with LidA. We also tested such mutants in other Rabs and did not observe an effect. So it remains undetermined how LidA selects different Rabs. Nonetheless, it is expected that the mechanism of Rab1 recognition by LidA is applicable to other Rab members given the high conservation in the LidA-interacting surface.

## Discussion

The crystal structures presented in current study revealed that the GDP-bound Rab1 is held in the active conformation through associating with LidA. Consistent with this structural observation, quantification assay using ITC demonstrated that LidA binds strongly to both GDP- and GTP-Rab1 with a nearly equal affinity (Figures 1A, B). These results indicate that Rab1 recognition by LidA is independent of its cycling between GDP- and GTP-bound states. To LidA, the switch mechanisms of Rab1 are not operational and thus Rab1 is rendered persistently active in interaction with LidA. Such a unique function of LidA provides explanations for a body of previous observations.

The prenylated GDI-free Rab1(D44N) was shown to be primarily membrane-localized, indicating that GDI is not absolutely required for membrane targeting of Rab1 [57]. This principle is likely applicable to the delivery of Rab1(D44N) to the LCV [45]. The strong interaction with LidA can be important for the membrane retention of Rab1(D44N), as LidA-deficient mutants displayed a scattered distribution of this Rab1 mutant in COS-1 cells [45]. Because no GDP-Rab1(D44N)-GDI complex is formed and LidA is able to recognize GDP-Rab1, GDF and GEF activity would be made redundant under these conditions, providing an explanation why recruitment of Rab1(D44N) to LCV is independent of SidM/DrrA [45]. To wild type Rab1, the complex of GDP-Rab1-GDI is formed with a high affinity. The direct competition of LidA with GDI for binding GDP-Rab1 will be difficult and inefficient. Thus GDP-Rab1 has to be released from GDI by SidM/DrrA for LidA recognition [39]. These arguments likely hold true with the recruitment of the GDP-restricted Rab1 mutant (S25N) to the LCV. In uninfected cells, the dominant inhibitory effect of Rab1(S25N) derives from the competition of this Rab1 mutant with wild type Rab1 for binding GEFs, resulting in formation of “dead-end” Rab1-GEF complexes [58]. In the case of wild type Rab1 recruitment to the LCV, this situation could be altered, because interaction of GDP-bound Rab1-SidM/DrrA complex is transient and much weaker than that of GDP-bound Rab1-LidA complex [39], [45] (Figure S3). LidA is therefore expected to be able to outcompete with SidM/DrrA for binding GDP-Rab1, which in turn facilitates the release of GDP-Rab1 from GDF (see further discussion below). As GDP-Rab1 is in the active conformation when complexed with LidA, it is conceivable that Rab1(S25N) can still support the functions performed by wild type Rab1. In complete agreement with this hypothesis, Rab1 binding to the LCV precedes the delivery of Sec22b, GDP-locked Rab1(S25N) can delay but not block the recruitment of Sec22b. This Rab1 mutant can support growth of *L. pneumophila*, at least for the first 10 hours of postinfection [42].

The strong association with LidA can facilitate the recruitment of Rab1 to LCV by SidM/DrrA. Release of GDP-Rab1 from GDIs catalyzed by SidM/DrrA (as a GDF) is a dynamic process and GDP-Rab1-SidM/DrrA is an intermediate of the reaction [59]–[61]. LidA is expected to displace SidM/DrrA from the intermediate because of its much higher binding affinity to GDP-Rab1, thus shifting the equilibrium toward dissociation of GDP-Rab1-GDI complex. In this respect, LidA is similar to GTP in that the latter is able to exchange GDP from the intermediate and form the GTP-bound Rab1, a product that is unfavorable for interaction with GDFs and thereby promotes disruption of GDP-Rab1-GDI complex. It can be imagined that lack of LidA would slow down the release of GDP-Rab1 from GDI. Consistently, deletion of LidA resulted in delayed but not a blocked recruitment of Rab1 to the LCV [39]. The inability of LidA to discriminate between GDP- and GTP-bound Rab1 may also be advantageous for *L. pneumophila* to retain the recruited Rab1 on the LCV. Because even GTP-bound Rab1 is subjected to hydrolysis by GAPs on LCV [43], [44], the resulting product of GDP-Rab1 is still able to be captured by LidA with a strong binding affinity. Moreover, interaction with LidA would make GDP-Rab1 less susceptible to extraction from the membrane of LCV by GDIs, because they have to outcompete LidA for Rab1 binding.

Unlike other small GTPase effector proteins which mainly binding to Rab1 through interacting with the switch I and II regions that determine nucleotide-dependent interaction, LidA holds Rab1 tightly in its hand like structure with four fingers. It is unlikely that any Rab1 effector can displace LidA from Rab1-LidA complex. LidA has been known to associate with the LCVs for much longer time than SidM/DrrA. However, Rab1 does eventually leave the LCV [44], [48], so that a mechanism is needed to decrease the binding affinity between Rab1 and LidA. Since Rab1 is deeply buried in the complex, the most efficient way to release Rab1 is to force LidA to open its fingers by downstream proteins. The GTP-Rab1-LidA structure shows truncation of the middle and the ring fingers does not disrupt Rab1-LidA complex, so that the thumb or the index fingers, though necessary, may not be sufficient to release Rab1 (Figure 4A). Structure analysis indicates that the thumb and the ring finger do not pack as tightly with other part of LidA as the index and middle finger. This means that LidA's wrist (the N-terminal half of α4 and β1–β4) which is isolated from the central interacting region, the bottom of the ring finger and thumb are accessible for other proteins to trigger a conformational change that releases Rab1 (Figure S4). Opening of the ring finger could be very important since it can expose the switch region immediately to facilitate Rab1 binding by other effectors.

Recent research showed that the post-translationally modified Rab1b by the *L. pneumophila* effector protein SidM/DrrA retains the ability to interact with LidA and can avoid the GAP recognition [46]. Our data confirmed that the correspondent residue Tyr80 in Rab1a was also AMPylated (Figure S5A). Tyr80 is located on the interface between Rab1a and LidA, involved in both hydrogen bonding with His431^LidA^ and hydrophobic contact with Leu406^LidA^ (Figure 3C), so it is reasonable to speculate that the covalent-bound AMP group on Tyr80 will affect the stability of LidA-Rab1 complex. However, Rab1 interacts with the LidA palm tightly via an extensive surface, so that the interaction is difficult to interrupt. In the structure of GTP-Rab1-LidA we can see even the middle and the ring fingers are truncated, LidA can still holds Rab1 (Figure 4A). Moreover the structural feature of four fingers possesses a certain degree of flexibility. Thus the orientations of the four fingers could adjust a little bit to accommodate the additional AMP group. Consistent with previous reports [46] our ITC results showed that the binding affinity of Rab1a(K62H, 1-176) and LidA(188-580) is not significantly affected by the AMPylation (Figure S5B,C). Thus we presume that LidA can form both the Rab1-LidA and AMP-Rab1-LidA complex in vivo. The ability of LidA to form stable complex with AMPylated Rab1 is consistent with its ability to bind a number of different Rabs, which small differences in their surface residues. *L. pneumophila* may also benefit from this property. Formation of AMPylated-Rab1-LidA complex may protect Rab1 from the de-AMPylation activity of SidD until Rab1 is released from LidA by still unknown mechanisms.

In addition to promoting SidM/DrrA for Rab1 recruitment, LidA may have other function(s). LidA interacts with the active conformation of Rab1 and exhibits no enzymatic activity [39]. Structural comparison showed that binding of LidA to Rab1 shares some features of the interaction between the effector protein Rabenosyn-5 with Rab4 [54]. Furthermore, recruitment of Rab1 is essential for delivering ER-derived vesicles to LCV [42], [43], [62]. Collectively, these results suggest that one potential function of LidA is to act as an effector of Rab1 and signal downstream components for remodeling of LCV. LidA was previously predicted and further confirmed in the present study to be a coiled-coil rich protein, one type of the best characterized Rab tethering factors [27], [54], [63]. The terminal parallel helix forms an antiparallel long coiled-coil and extends to the outside of interacting region (Figure 2C). Secondary structure analysis suggests that in full-length LidA, the length of this domain is likely to be longer than 100 Å. It is thus possible that LidA may function as a tethering factor, bridging the ER-derived vesicles and the LCV [49]. One potential advantage of employing its own protein as a tethering factor would allow *L. pneumophila* to be selective for recruiting ER-derived components for its growth. It is well established that a GTP-bound form of Rab GTPases interacts with their effectors for signaling. The only exception to this rule is Protrudin that interacts with the GDP-bound form of Rab11, regulating Rab11-dependent membrane recycling to promote the directional membrane trafficking [64]. While interacting with GDP-Rab1, LidA appears to be different from protrudin in that it essentially recognizes the active conformation of Rab1 and probably other Rabs [39], [43].

During the peer review of this manuscript, the structure of Rab8-LidA complex was published online. Structure comparison shows that Rab8-LidA complex adopts quite similar conformations with our structure and that the binding interface is highly conserved, consistent with our proposal that lidA recognizes Rabs in a similar way. We note that the authors report a binding affinity of LidA for GTP-bound Rab1 that is roughly one order of magnitude stronger than what we measured using ITC. At this point, it is inconsistent with our ITC data that LidA binds to both GDP- and GTP-Rab1 with a nearly equal affinity.

## Materials and Methods

### Protein expression purification

The genes of *lidA* and *sidM* were amplified from genomic DNA of *Legionella pneumophila* strain Corby. The DNA fragment encoding human Rab1a (noted Rab1 throughout the text) and other 14 kinds of Rab family members were amplified from a homemade human cDNA library. All the genes encoding the mutant proteins were produced using a two-step PCR procedure. LidA, both full-length (FL) and various fragments, SidM variants include 317-545 and 1-545 were subcloned into the pET15b (Invitrogen), Rab1 variants and Rab2, 4, 6, 7, 8, 11, 14, 18, 23, 27 were subcloned into the pGEX-6P-1 plasmid (GE Healthcare), Rab5, 9, 20, 22 were subcloned into both of the two vectors. Each protein was produced in *E. coli* BL21 (DE3). Cells grow at 37°C until the OD_600_ reach to 0.8. LidA proteins were induced with 0.5 mM isopropyl- β-D-Thiogalactopyranoside (IPTG) for 15 h at 15°C, Rab1 and other Rabs were all induced with 0.2 mM IPTG for 15 h at 22°C. All proteins were purified using affinity, anion exchange and gel filtration chromatography. To obtain LidA-Rab1 complexes, LidA and Rab1 proteins were mixed up with a 1∶1 molar ratio at 4°C for 2 hours. The LidA(188-449)-Rab1(1-191) complex and LidA(224-559)-Rab1(S25N; 1-176) complex were further purified using the above mentioned procedure except the GST tag and His tag were removed by homemade PreScission protease digestion before the anion exchange step. Both of the protein complexes have a final concentration of 10 mg/mL. All purification processes were performed at 4°C unless noted otherwise.

### Crystallization and data collection

Crystallization conditions for complexes were determined from the sparse matrix screen (Hampton Research). All crystals were obtained using the hanging drop diffusion method at 20°C. LidA(224-559)-Rab1(S25N, 1-176) complex was crystallized by mixing equal volumes of protein with reservoir solution containing 28% Jeffamin ED-2001 and 0.1 M sodium citrate tribasic dihydrate pH 4.8. LidA(224-559)-Rab1(S25N, 1-176) crystals were optimized by microseeding to reach the satisfied diffraction. LidA(188-449)-Rab1(wild-type, WT1-191) complex was crystallized by mixing equal volumes of protein with reservoir solution containing 0.1 M Hepes pH 7.5, 25% PEG3350 and 0.7% butanol. All diffraction data were collected at Shanghai Synchrotron Radiation facility (SSRF) beamline BL17U. To prevent radiation damage, crystals were equilibrated in a cryoprotectant buffer containing 20% ethylene glycol (v/v) plus reservoir buffer and then flash frozen in a 100 K nitrogen stream. The best crystal of LidA(225-559)-Rab1(S25N; 1-176) complex diffracted to 1.73 Å. The best crystal of LidA(188-449)-Rab1(WT; 1-191) complex diffracted to 2.2 Å. Data sets were processed using the HKL2000 software suite [65].

### Structure determination

The crystal structures of the two kinds of LidA-Rab1 complexes were determined by molecular replacement using PHASER [66] with the coordinates of Rab1 as the searching models (PDB ID code 2FOL) [67]. The atomic models were built using COOT [68] and refined using PHENIX [69]. Data collection and structure refinement statistics are summarized in Table S1. A SA-composite omit map of LidA index finger shows the good match between the atom skeleton and the electron density map as supplementary Figure S6. All the molecular graphics Figures were generated using PyMol (http://www.pymol.org).

### Isothermal titration calorimetry

ITC was employed to measure the binding affinities of various fragments of LidA with Rab1 variants and 9 kinds of Rabs. All protein samples were purified in a buffer containing 20 mM Hepes (pH 8.0) and 100 mM NaCl with tag removed by PreScission protease digestion. The final concentration of LidA(188-580) and LidA(188-449) were 0.2 mM, Rab1(S25N) and Rab1(Q70L) were 2 mM, LidA(FL) was 0.15 mM, Rabs(FL) (include 2, 4, 6, 7, 9, 11, 20, 22) were 1.5 mM. The samples were centrifuged to remove any precipitate before the experiments. All measurements were carried out at 25°C by using a VP-ITC microcalorimeter 200 (MicroCal). Titrations were carried out by titrating Rab1(S25N) and Rab1(Q70L) into LidA(188-580) or LidA(188-449), respectively. Rab2, 4, 6, 7, 9, 11, 20 proteins were titrated into LidA(188-580). Rab22 were titrated into LidA(FL). The titration Data were analyzed using ORIGIN data analysis software (MicroCalSoftware).

### *In vitro* pull down assay

15 kinds of purified Rab family members (WT; FL) with N-terminal GST tag (His-fused Rab20 is exceptional) was immobilized onto 200 µL glutathione-Sepharose resin (GE) or Ni-chelating sepharose (GE healthcare) for His-Rab20. The resin was then washed three times to remove excess unbound protein. Untagged LidA(FL) protein solution was loaded onto the Rabs-immobilized beads and incubated at 4°C for 2 h. The loaded protein dose of LidA and Rabs was at a molar ratio of 2∶1. The resin was washed three times using the wash buffer (25 mM Tris-HCl buffer pH 8.0, 100 mM NaCl) to remove unbound LidA. Proteins binding to the resin were eluted by elution buffer (25 mM Tris-HCl buffer pH 8.0, 100 mM NaCl, 3 mM DTT, 5 mM GSH for glutathione-Sepharose resin and 25 mM Tris-HCl buffer pH 8.0, 100 mM NaCl, 250 mM imidazole for Ni-chelating sepharose). All samples were subjected to SDS-PAGE which was visualized by Coomassie Brilliant Blue staining.

### Accession numbers

Crystal structure of LidA(224-559)-Rab1(S25N; 1-176) complex and the structure factor have been deposited in the Protein Data Bank (http://www.rcsb.org/pdb) under ID codes 3SFV. Crystal structure of LidA(188-449)-Rab1(Q70L; 1-191) complex and the structure factor are deposited with access codes 3TKL.

## Supporting Information

Figure S1 **Domain mapping of LidA.** (A) Schematic representation of LidA fragments used in minimal domain mapping assay, numbers indicate amino acid residues, the corresponding binding affinity are labeled by plus/minus marker. (B) Gel filtration assay for LidA fragments-Rab1 complex formation. The proteins of Rab1(Q70L), LidA(FL) and variants were subject to gel filtration. Aliquots of the peak fraction corresponding to the position of LidA-Rab1 complex were subjected to SDS-PAGE which were visualized by Coomassie Brilliant Blue staining.
(TIF)

Figure S2 **Representation of 5 related GTP or GTP analogue-bound Rab GTPase structures.** (A) Left: cartoon representation of the GTP-bound Rab1a(residues 1-176) structure from the LidA(188-449)-Rab1(WT; 1-191) complex. Right: the hydrogen bonds between GTP phosphate group and Gly69, Thr43 in the structure of GTP-bound Rab1 complexed with LidA. The GTP-Rab1 is shown in pink; the switch I, switch II and P-loop are shown in red, purple and green, respectively. (B–E) Cartoon representation of GppNHp-bound Ypt1 (PDB ID CODE 1YZN) (B), GTP-bound Rab4 (PDB ID CODE 1Z0K) (C), GTP-bound Rab6 (PDB ID CODE 2GIL) (D) and GTP-bound Rab7 (PDB ID CODE 1T91) (E), respectively. The conserved hydrogen bonding interactions among GTP (or GTP analogue GppNHp) and switch regions are denoted on the structure by black dashed lines. All structures are shown in the same orientation. Switch I, Switch II, P-loop and Mg^2+^ are indicated by the colors as shown. GTP (or GTP analogue GppNHp) are shown as sticks, Mg^2+^ is shown as sphere, the length of the conserved hydrogen bonds are indicated.
(TIF)

Figure S3 **SidM(317-545)-Rab1(1-176) complex disassociation induced by LidA and nucleotides.** The SidM(317-545)-Rab1(1-176) complex was mixed with LidA(191-600) at a molar ratio of 1∶5 and subjected to gel filtration in presence of 5 mM GDP (gray lines) or GTP (black lines); The same mixture in absence of nucleotides is shown as red lines. All samples were analyzed by size exclusion chromatography on a Superdex-200 column, monitored by UV absorption at 280 nm, the peak elution volumes are labeled as shown. The formation of LidA(191-600)-Rab1(1-176) complexes were observed only in presence of the nucleotides.
(TIF)

Figure S4 **LidA potential interface for downstream effectors.** Cartoon representation of the LidA structural interface to potential effectors. Rab1(S25N) and the switch regions, and LidA's fingers are color-coded the same as Figure 2. Double arrow points to the LidA-Rab1 interacting gab and switch regions, respectively. LidA's potential structural interaction domains with downstream effector exist in two regions, labeled as the arrow point. Since the key interaction regions with cellular ligands of Rab1 known as switch I and II are occupied by LidA and buried inside the complex, it is hard for other Rab effectors to replace the LidA for Rab1 binding, thus the bottom of the ring finger in LidA, labeled as “LidA interaction region 1”, and the wrist (the N-terminal half of α4 and β1–β4) which is isolated from the central interacting region, labeled as “LidA interaction region 2”, are accessible for other proteins to bind with LidA and may cause allosteric effect on these fingers to release Rab1.
(TIF)

Figure S5 **Rab1 AMPylation cause no effect to LidA-binding ability** ***in vitro*** **.** (A) Mass spectrometry analysis of Rab1a(K62H,1-176) show LidA has no influence on Rab1a AMPylation reaction *in vitro*. MALDI-TOF reflector spectra of tryptic digestion of three samples. (Top) Unmodified Rab1a(K62H,1-176), this mutant can eliminate the 1317 daltons mass peak _62_LQIWDTAGQER_72_ in native Rab1 which cause superposition with the peak of the AMPylated _75_TITSSY-_AMP_YR_82_ peptide (1319 daltons). (Middle) AMPylated Rab1a(K62H,1-176) treated by SidM(1-545) for 4 h. The inset in zoom showing the intensity of _75_TITSSYYR_82_ peptide at m/z 990, and the existence of AMPylated peptide _75_TITSSY-_AMP_YR_82_ at m/z 1319. (Bottom) AMPylated Rab1a(K62H,1-176) treated by both SidM(1-545) and LidA(FL). Both peptide at m/z 1319 and m/z 990 have no significant mass shift compared with the one shown in middle mass spectrum Figure. The insets show magnification of the peaks around m/z 990 and 1319. (B) Measurement of binding affinity between LidA(188-580) and unmodified Rab1a(K62H,1-176) by ITC. Top panel: twenty injections of Rab1a(K62H,1-176) solutions were titrated into LidA(188-580) solution ITC cell. The area of each injection peak corresponds to the total heat released for that injection. Bottom panel: the binding isotherm for Rab1a(K62H,1-176) and LidA(188-580) interaction, the integrated heat is plotted against the stoichiometry in 1∶1, data fitting revealed a binding affinity as shown. (C) Measurement of binding affinity between LidA(188-580) and AMPylated Rab1a(K62H,1-176) by ITC, the experiment conditions was exactly the same as B unless the Rab1a(K62H,1-176) is AMPylated before the titration.
(TIF)

Figure S6 **Representative the composite omit map of LidA index finger.** The SA-composite map of LidA(385-455) in the LidA-Rab1(S25N) complex. This part is a bit longer than the index finger (from 387 to 449), the corresponding sequence is shown. The Figure demonstrates the quality of the electron density (blue mesh). Both of the electron densities are depicted at 1.5 σ.
(TIF)

Protocol S1 **Domain mapping by gel filtration.** To map the interaction domain of LidA with Rab1, the proteins of LidA(FL) and fragments, Rab1(FL, Q70L) were purified by affinity chromatography with N-terminal His-tag, then mixed protein solutions together at 4°C for 2 h, the protein concentration of Rab1 and LidA was at a molar ratio of 1∶1. Afterwards, the protein mixtures were subject to size exclusion chromatography on a Superdex-200, monitored by UV absorption at 280 nm. The buffer of gel filtration containing 25 mM Tris-HCl (pH 8.0), 100 mM NaCl, and 3 mM DTT. Each aliquots of the peak fraction were subjected to SDS-PAGE which were visualized by Coomassie Brilliant Blue staining.
(DOCX)

Protocol S2 **In vitro AMPylation of Rab1a(K62H, 1-176) and purification of AMP-Rab1a(K62H, 1-176).** Rab1a(K62H,1-176) protein was AMPylated in the presence of 2.5 molar excess of ATP and 0.01 molar ratio of SidM at room temperature for 4 h, afterwards, the AMPylated Rab1a(K62H,1-176) was purified by gel filtration on a Superdex-200 10/300 column (GE healthcare) at 4°C. Fractions containing AMPylated Rab1a(K62H,1-176) in 20 mM Hepes (pH 8.0), 100 mM NaCl were pooled, concentrated to 1.5 mM, and stored at −80°C. Completeness of AMPylated Rab1a was verified by mass spectrometry as described in mass spectrometry.
(DOCX)

Protocol S3 **Mass spectrometry.** Rab1a(K62H,1-176) protein was separated by SDS-PAGE gel, and Coomassie brilliant blue stained. The in-gel digestion of Rab1a(K62H,1-176) for mass spectrometric analysis was performed as published previously [1]. Peptides were dissolved with 0.5% trifluoroacetic acid from digest mixture, and peptide mass analysis was performed using AB4700 MALDI-TOF/TOF mass spectrometer (Applied Biosystems). All data were acquired in the positive ion mode over an m/z range of 500–2000 Da.
(DOCX)

Protocol S4 **Isothermal titration calorimetry.** ITC was employed to measure the binding affinities of LidA(188-580) with Rab1a(K62H,1-176) or AMPylated Rab1a(K62H,1-176). All protein samples were purified in a buffer containing 20 mM Hepes (pH 8.0) and 100 mM NaCl. The final concentration of LidA(188-580) were 0.12 mM, Rab1a(K62H,1-176) and AMPylated Rab1a(K62H,1-176) were 1.5 mM. The samples were centrifuged to remove any precipitate before the experiments. Both titrations were performed in the absence of added Mg^2+^ and ATP. All measurements were carried out at 25°C by using a VP-ITC microcalorimeter 200 (MicroCal). Titrations were carried out by titrating Rab1a(K62H,1-176) or AMPylated Rab1a(K62H,1-176) into LidA(188-580), respectively; The titration Data were analyzed using ORIGIN data analysis software (MicroCalSoftware).
(DOCX)

Table S1 **Data collection and structure refinement statistics.**
(DOCX)

## References

[1] Zerial M, McBride H (2001). Rab proteins as membrane organizers.. Nat Rev Mol Cell Biol.

[2] Pfeffer SR (2001). Rab GTPases: specifying and deciphering organelle identity and function.. Trends Cell Biol.

[3] Segev N (2001). Ypt and Rab GTPases: insight into functions through novel interactions.. Curr Opin Cell Biol.

[4] Seabra MC, Wasmeier C (2004). Controlling the location and activation of Rab GTPases.. Curr Opin Cell Biol.

[5] Ullrich O, Stenmark H, Alexandrov K, Huber LA, Kaibuchi K (1993). Rab GDP dissociation inhibitor as a general regulator for the membrane association of rab proteins.. J Biol Chem.

[6] Ullrich O, Horiuchi H, Bucci C, Zerial M (1994). Membrane association of Rab5 mediated by GDP-dissociation inhibitor and accompanied by GDP/GTP exchange.. Nature.

[7] Pfeffer SR, Dirac-Svejstrup AB, Soldati T (1995). Rab GDP dissociation inhibitor: putting rab GTPases in the right place.. J Biol Chem.

[8] Dirac-Svejstrup AB, Sumizawa T, Pfeffer SR (1997). Identification of a GDI displacement factor that releases endosomal Rab GTPases from Rab-GDI.. EMBO J.

[9] Pereira-Leal JB, Hume AN, Seabra MC (2001). Prenylation of Rab GTPases: molecular mechanisms and involvement in genetic disease.. FEBS Lett.

[10] Calero M, Chen CZ, Zhu W, Winand N, Havas KA (2003). Dual prenylation is required for Rab protein localization and function.. Mol Biol Cell.

[11] Gomes AQ, Ali BR, Ramalho JS, Godfrey RF, Barral DC (2003). Membrane targeting of Rab GTPases is influenced by the prenylation motif.. Mol Biol Cell.

[12] Siniossoglou S, Peak-Chew SY, Pelham HR (2000). Ric1p and Rgp1p form a complex that catalyses nucleotide exchange on Ypt6p.. EMBO J.

[13] Rossman KL, Der CJ, Sondek J (2005). GEF means go: turning on RHO GTPases with guanine nucleotide-exchange factors.. Nat Rev Mol Cell Biol.

[14] Bos JL, Rehmann H, Wittinghofer A (2007). GEFs and GAPs: critical elements in the control of small G proteins.. Cell.

[15] Barr F, Lambright DG (2010). Rab GEFs and GAPs.. Curr Opin Cell Biol.

[16] McLauchlan H, Newell J, Morrice N, Osborne A, West M (1998). A novel role for Rab5-GDI in ligand sequestration into clathrin-coated pits.. Curr Biol.

[17] Koumandou VL, Dacks JB, Coulson RM, Field MC (2007). Control systems for membrane fusion in the ancestral eukaryote; evolution of tethering complexes and SM proteins.. BMC Evol Biol.

[18] Batoko H, Zheng HQ, Hawes C, Moore I (2000). A rab1 GTPase is required for transport between the endoplasmic reticulum and golgi apparatus and for normal golgi movement in plants.. Plant Cell.

[19] Muslin AJ (2001). Road Rage: Cardiac Rab1 and ER-to-Golgi Traffic.. Circ Res.

[20] Filipeanu CM, Zhou F, Claycomb WC, Wu G (2004). Regulation of the cell surface expression and function of angiotensin II type 1 receptor by Rab1-mediated endoplasmic reticulum-to-Golgi transport in cardiac myocytes.. J Biol Chem.

[21] Grosshans BL, Ortiz D, Novick P (2006). Rabs and their effectors: achieving specificity in membrane traffic.. Proc Natl Acad Sci U S A.

[22] Haas AK, Yoshimura S, Stephens DJ, Preisinger C, Fuchs E (2007). Analysis of GTPase-activating proteins: Rab1 and Rab43 are key Rabs required to maintain a functional Golgi complex in human cells.. J Cell Sci.

[23] Yin H, Li Q, Qian G, Wang Y, Li Y (2011). Rab1 GTPase regulates phenotypic modulation of pulmonary artery smooth muscle cells by mediating the transport of angiotensin II type 1 receptor under hypoxia.. Int J Biochem Cell Biol.

[24] Allan BB, Moyer BD, Balch WE (2000). Rab1 recruitment of p115 into a cis-SNARE complex: programming budding COPII vesicles for fusion.. Science.

[25] Moyer BD, Allan BB, Balch WE (2001). Rab1 interaction with a GM130 effector complex regulates COPII vesicle cis–Golgi tethering.. Traffic.

[26] Beard M, Satoh A, Shorter J, Warren G (2005). A cryptic Rab1-binding site in the p115 tethering protein.. J Biol Chem.

[27] Sztul E, Lupashin V (2006). Role of tethering factors in secretory membrane traffic.. Am J Physiol Cell Physiol.

[28] Albert-Weissenberger C, Cazalet C, Buchrieser C (2007). Legionella pneumophila - a human pathogen that co-evolved with fresh water protozoa.. Cell Mol Life Sci.

[29] Horwitz MA (1983). Formation of a novel phagosome by the Legionnaires' disease bacterium (Legionella pneumophila) in human monocytes.. J Exp Med.

[30] Sturgill-Koszycki S, Swanson MS (2000). Legionella pneumophila replication vacuoles mature into acidic, endocytic organelles.. J Exp Med.

[31] Ensminger AW, Isberg RR (2009). Legionella pneumophila Dot/Icm translocated substrates: a sum of parts.. Curr Opin Microbiol.

[32] Derre I, Isberg RR (2004). Legionella pneumophila replication vacuole formation involves rapid recruitment of proteins of the early secretory system.. Infect Immun.

[33] Bruggemann H, Cazalet C, Buchrieser C (2006). Adaptation of Legionella pneumophila to the host environment: role of protein secretion, effectors and eukaryotic-like proteins.. Curr Opin Microbiol.

[34] Ninio S, Roy CR (2007). Effector proteins translocated by Legionella pneumophila: strength in numbers.. Trends Microbiol.

[35] Shin S, Roy CR (2008). Host cell processes that influence the intracellular survival of Legionella pneumophila.. Cell Microbiol.

[36] Isberg RR, O'Connor TJ, Heidtman M (2009). The Legionella pneumophila replication vacuole: making a cosy niche inside host cells.. Nat Rev Microbiol.

[37] Wu G (2008). Regulation of the trafficking and function of G protein-coupled receptors by Rab1 GTPase in cardiomyocytes.. Methods Enzymol.

[38] Zhuang X, Adipietro KA, Datta S, Northup JK, Ray K (2010). Rab1 small GTP-binding protein regulates cell surface trafficking of the human calcium-sensing receptor.. Endocrinology.

[39] Machner MP, Isberg RR (2006). Targeting of host Rab GTPase function by the intravacuolar pathogen Legionella pneumophila.. Dev Cell.

[40] Murata T, Delprato A, Ingmundson A, Toomre DK, Lambright DG (2006). The Legionella pneumophila effector protein DrrA is a Rab1 guanine nucleotide-exchange factor.. Nat Cell Biol.

[41] Brumell JH, Scidmore MA (2007). Manipulation of rab GTPase function by intracellular bacterial pathogens.. Microbiol Mol Biol Rev.

[42] Kagan JC, Stein MP, Pypaert M, Roy CR (2004). Legionella subvert the functions of Rab1 and Sec22b to create a replicative organelle.. J Exp Med.

[43] Neunuebel MR, Chen Y, Gaspar AH, Backlund PS, Yergey A (2011). De-AMPylation of the small GTPase Rab1 by the pathogen Legionella pneumophila.. Science.

[44] Ingmundson A, Delprato A, Lambright DG, Roy CR (2007). Legionella pneumophila proteins that regulate Rab1 membrane cycling.. Nature.

[45] Machner MP, Isberg RR (2007). A bifunctional bacterial protein links GDI displacement to Rab1 activation.. Science.

[46] Muller MP, Peters H, Blumer J, Blankenfeldt W, Goody RS (2010). The Legionella effector protein DrrA AMPylates the membrane traffic regulator Rab1b.. Science.

[47] Tan Y, Luo ZQ (2011). Legionella pneumophila SidD is a deAMPylase that modifies Rab1.. Nature.

[48] Conover GM, Derre I, Vogel JP, Isberg RR (2003). The Legionella pneumophila LidA protein: a translocated substrate of the Dot/Icm system associated with maintenance of bacterial integrity.. Mol Microbiol.

[49] Derre I, Isberg RR (2005). LidA, a translocated substrate of the Legionella pneumophila type IV secretion system, interferes with the early secretory pathway.. Infect Immun.

[50] Nuoffer C, Davidson HW, Matteson J, Meinkoth J, Balch WE (1994). A GDP-bound of rab1 inhibits protein export from the endoplasmic reticulum and transport between Golgi compartments.. J Cell Biol.

[51] Wilson BS, Nuoffer C, Meinkoth JL, McCaffery M, Feramisco JR (1994). A Rab1 mutant affecting guanine nucleotide exchange promotes disassembly of the Golgi apparatus.. J Cell Biol.

[52] Tisdale EJ, Bourne JR, Khosravi-Far R, Der CJ, Balch WE (1992). GTP-binding mutants of rab1 and rab2 are potent inhibitors of vesicular transport from the endoplasmic reticulum to the Golgi complex.. J Cell Biol.

[53] Stroupe C, Brunger AT (2000). Crystal structures of a Rab protein in its inactive and active conformations.. J Mol Biol.

[54] Eathiraj S, Pan X, Ritacco C, Lambright DG (2005). Structural basis of family-wide Rab GTPase recognition by rabenosyn-5.. Nature.

[55] Pfeffer SR (2005). Structural clues to Rab GTPase functional diversity.. J Biol Chem.

[56] Urwyler S, Nyfeler Y, Ragaz C, Lee H, Mueller LN (2009). Proteome analysis of Legionella vacuoles purified by magnetic immunoseparation reveals secretory and endosomal GTPases.. Traffic.

[57] Wilson AL, Erdman RA, Maltese WA (1996). Association of Rab1B with GDP-dissociation inhibitor (GDI) is required for recycling but not initial membrane targeting of the Rab protein.. J Biol Chem.

[58] Storrie B (2005). Microinjection as a tool to explore small GTPase function.. Methods Enzymol.

[59] Schoebel S, Oesterlin LK, Blankenfeldt W, Goody RS, Itzen A (2009). RabGDI displacement by DrrA from Legionella is a consequence of its guanine nucleotide exchange activity.. Mol Cell.

[60] Suh HY, Lee DW, Lee KH, Ku B, Choi SJ (2010). Structural insights into the dual nucleotide exchange and GDI displacement activity of SidM/DrrA.. EMBO J.

[61] Zhu Y, Hu L, Zhou Y, Yao Q, Liu L (2010). Structural mechanism of host Rab1 activation by the bifunctional Legionella type IV effector SidM/DrrA.. Proc Natl Acad Sci U S A.

[62] Brombacher E, Urwyler S, Ragaz C, Weber SS, Kami K (2009). Rab1 guanine nucleotide exchange factor SidM is a major phosphatidylinositol 4-phosphate-binding effector protein of Legionella pneumophila.. J Biol Chem.

[63] Markgraf DF, Peplowska K, Ungermann C (2007). Rab cascades and tethering factors in the endomembrane system.. FEBS Lett.

[64] Shirane M, Nakayama KI (2006). Protrudin induces neurite formation by directional membrane trafficking.. Science.

[65] Otwinowski Z MW (1997). Processing of X-ray Diffration Data Collected in Oscillation Mode.. Methods in Enzymology.

[66] McCoy AJ, Grosse-Kunstleve RW, Adams PD, Winn MD, Storoni LC (2007). Phaser crystallographic software.. J Appl Crystallogr.

[67] (1994). The CCP4 suite: programs for protein crystallography.. Acta Crystallogr D Biol Crystallogr.

[68] Emsley P, Cowtan K (2004). Coot: model-building tools for molecular graphics.. Acta Crystallogr D Biol Crystallogr.

[69] Adams PD, Grosse-Kunstleve RW, Hung LW, Ioerger TR, McCoy AJ (2002). PHENIX: building new software for automated crystallographic structure determination.. Acta Crystallogr D Biol Crystallogr.

