# Supplementary figures and images for: Structural Insights into a Unique Legionella pneumophila Effector LidA Recognizing Both GDP and GTP Bound Rab1 in Their Active State
Source: PLoS Pathog. 2012 Mar 1;8(3):e1002528. doi: 10.1371/journal.ppat.1002528 (PMC3295573; doi:10.1371/journal.ppat.1002528)

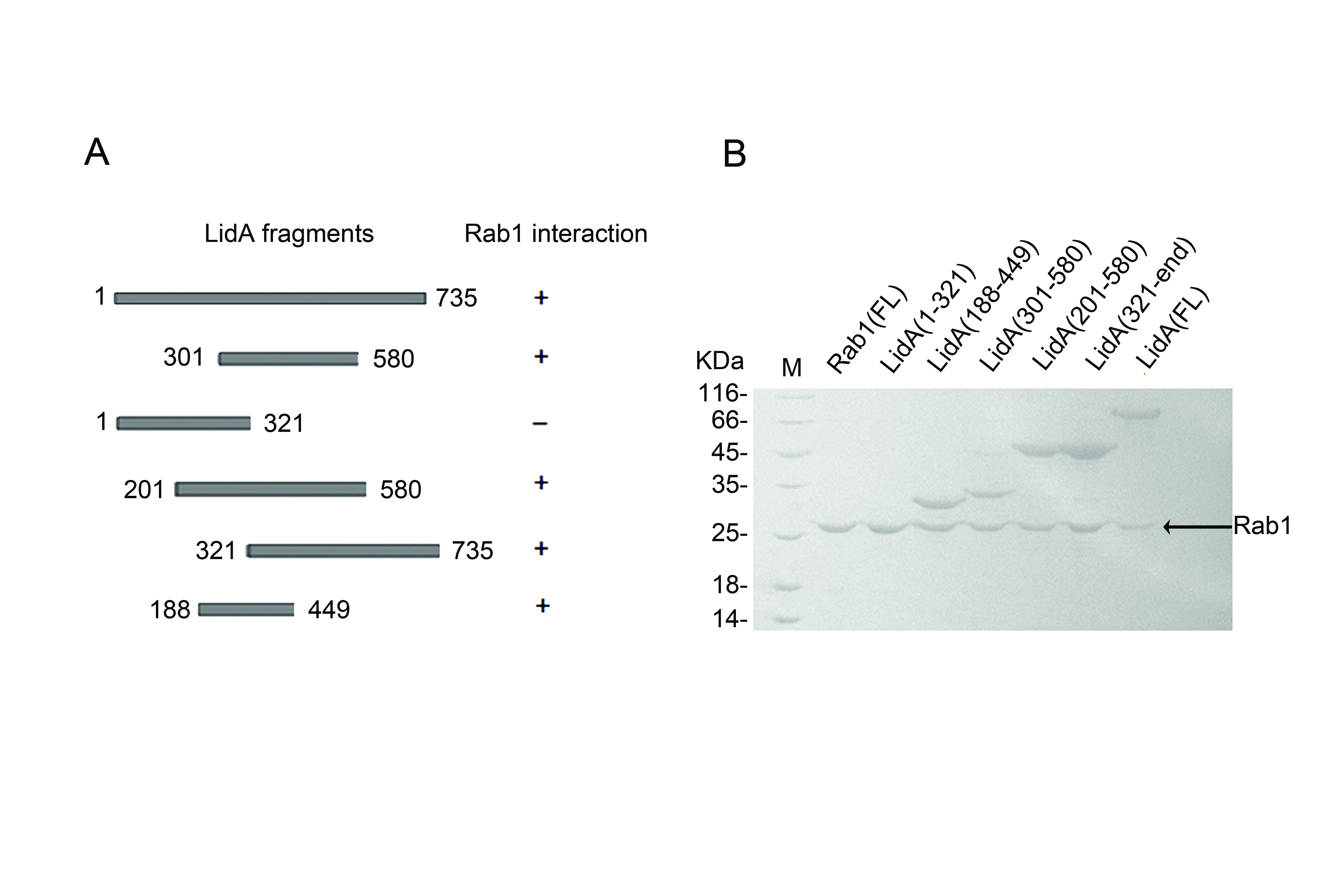

Supplement: Figure S1 — Domain mapping of LidA. (A) Schematic representation of LidA fragments used in minimal domain mapping assay, numbers indicate amino acid residues, the corresponding binding affinity are labeled by plus/minus marker. (B) Gel filtration assay for LidA fragments-Rab1 complex formation. The proteins of Rab1(Q70L), LidA(FL) and variants were subject to gel filtration. Aliquots of the peak fraction corresponding to the position of LidA-Rab1 complex were subjected to SDS-PAGE which were visualized by Coomassie Brilliant Blue staining. (TIF) [file ppat.1002528.s001.tif]

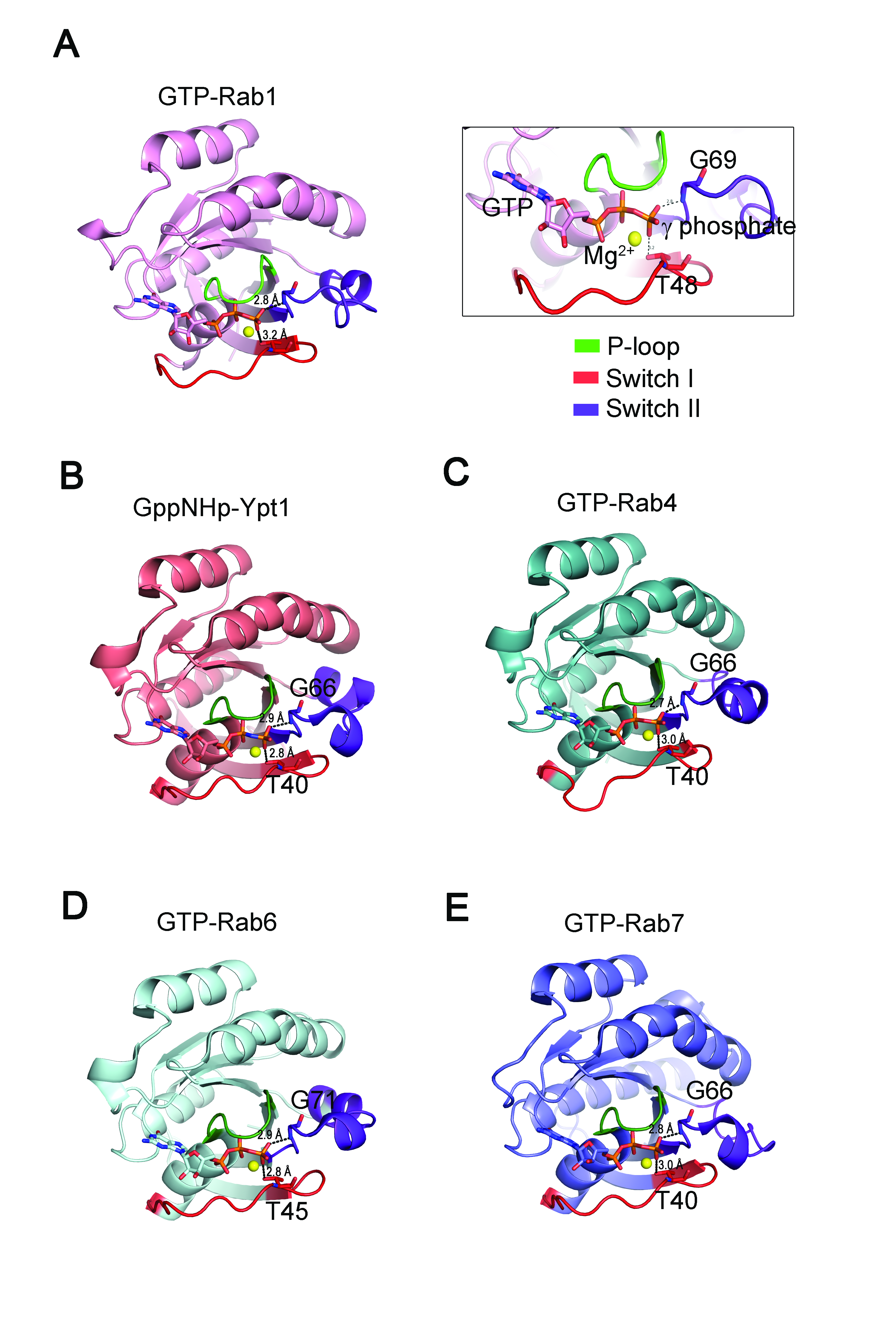

Supplement: Figure S2 — Representation of 5 related GTP or GTP analogue-bound Rab GTPase structures. (A) Left: cartoon representation of the GTP-bound Rab1a(residues 1-176) structure from the LidA(188-449)-Rab1(WT; 1-191) complex. Right: the hydrogen bonds between GTP phosphate group and Gly69, Thr43 in the structure of GTP-bound Rab1 complexed with LidA. The GTP-Rab1 is shown in pink; the switch I, switch II and P-loop are shown in red, purple and green, respectively. (B–E) Cartoon representation of GppNHp-bound Ypt1 (PDB ID CODE 1YZN) (B), GTP-bound Rab4 (PDB ID CODE 1Z0K) (C), GTP-bound Rab6 (PDB ID CODE 2GIL) (D) and GTP-bound Rab7 (PDB ID CODE 1T91) (E), respectively. The conserved hydrogen bonding interactions among GTP (or GTP analogue GppNHp) and switch regions are denoted on the structure by black dashed lines. All structures are shown in the same orientation. Switch I, Switch II, P-loop and Mg2+ are indicated by the colors as shown. GTP (or GTP analogue GppNHp) are shown as sticks, Mg2+ is shown as sphere, the length of the conserved hydrogen bonds are indicated. (TIF) [file ppat.1002528.s002.tif]

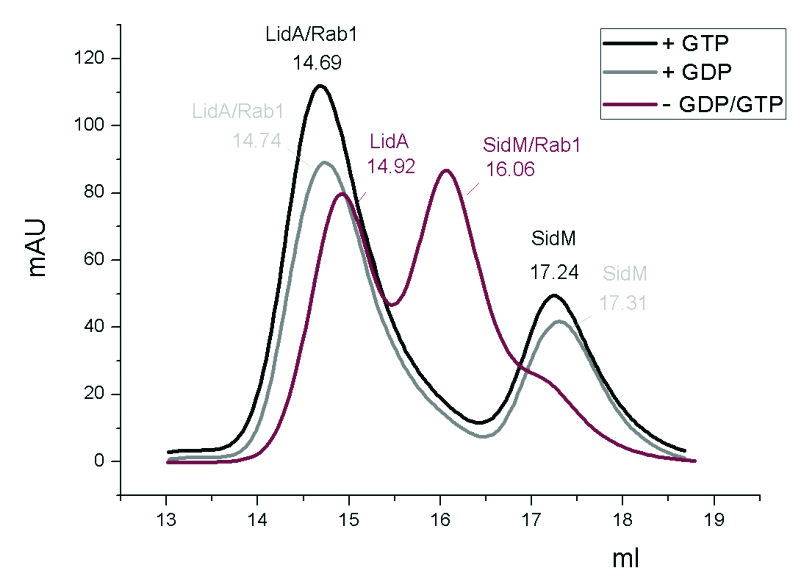

Supplement: Figure S3 — SidM(317-545)-Rab1(1-176) complex disassociation induced by LidA and nucleotides. The SidM(317-545)-Rab1(1-176) complex was mixed with LidA(191-600) at a molar ratio of 1∶5 and subjected to gel filtration in presence of 5 mM GDP (gray lines) or GTP (black lines); The same mixture in absence of nucleotides is shown as red lines. All samples were analyzed by size exclusion chromatography on a Superdex-200 column, monitored by UV absorption at 280 nm, the peak elution volumes are labeled as shown. The formation of LidA(191-600)-Rab1(1-176) complexes were observed only in presence of the nucleotides. (TIF) [file ppat.1002528.s003.tif]

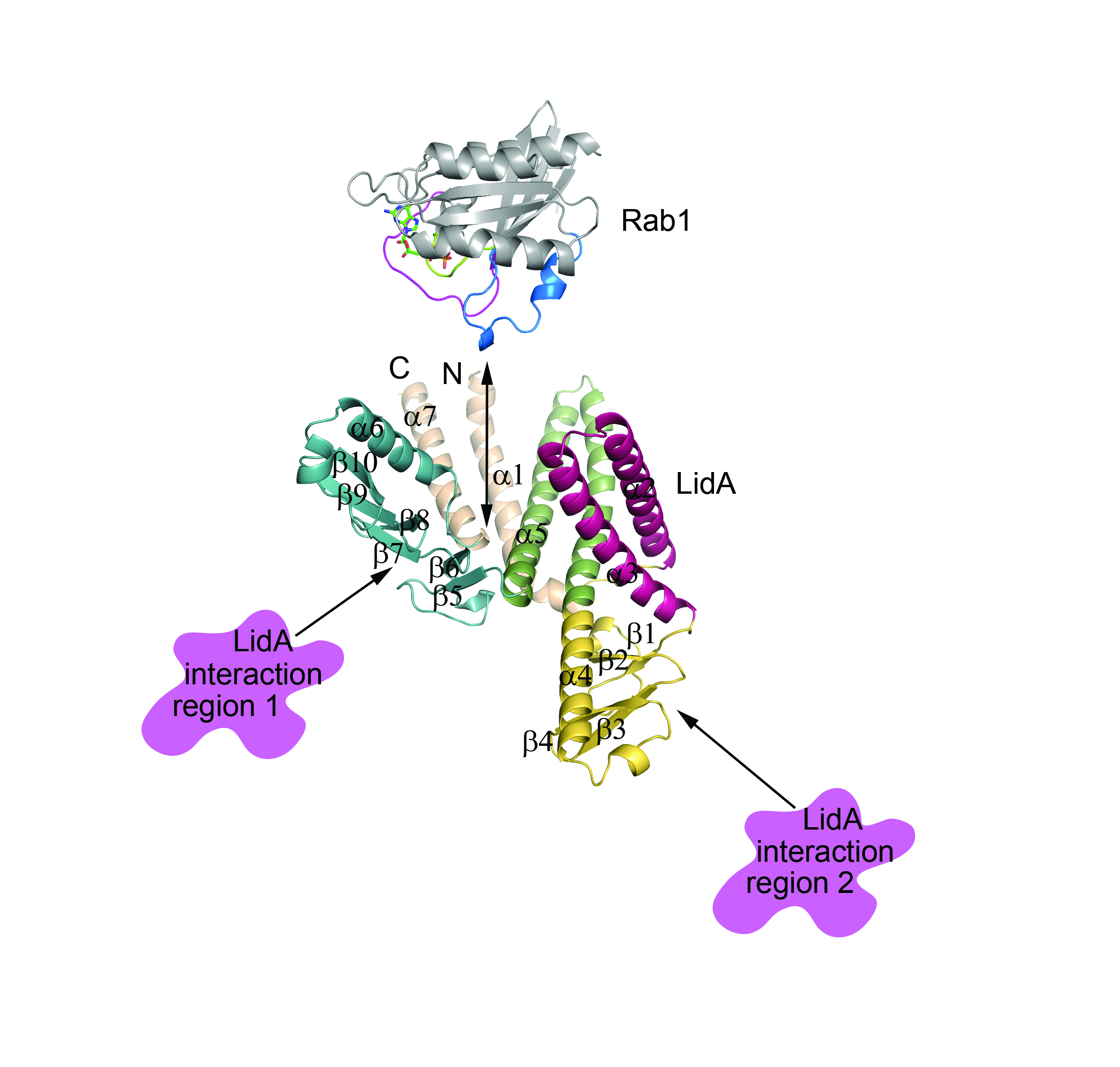

Supplement: Figure S4 — LidA potential interface for downstream effectors. Cartoon representation of the LidA structural interface to potential effectors. Rab1(S25N) and the switch regions, and LidA's fingers are color-coded the same as Figure 2. Double arrow points to the LidA-Rab1 interacting gab and switch regions, respectively. LidA's potential structural interaction domains with downstream effector exist in two regions, labeled as the arrow point. Since the key interaction regions with cellular ligands of Rab1 known as switch I and II are occupied by LidA and buried inside the complex, it is hard for other Rab effectors to replace the LidA for Rab1 binding, thus the bottom of the ring finger in LidA, labeled as “LidA interaction region 1”, and the wrist (the N-terminal half of α4 and β1–β4) which is isolated from the central interacting region, labeled as “LidA interaction region 2”, are accessible for other proteins to bind with LidA and may cause allosteric effect on these fingers to release Rab1. (TIF) [file ppat.1002528.s004.tif]

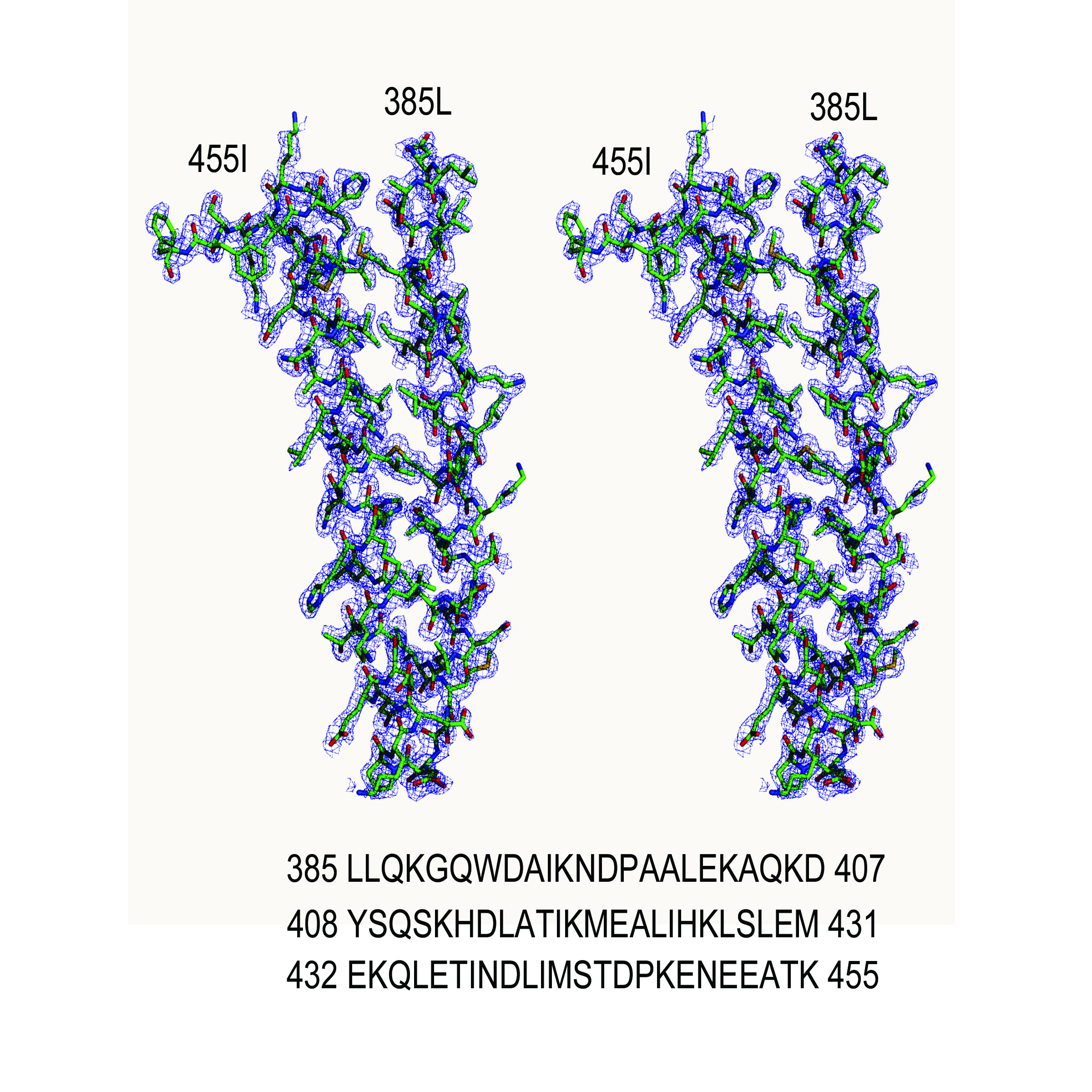

Supplement: Figure S6 — Representative the composite omit map of LidA index finger. The SA-composite map of LidA(385-455) in the LidA-Rab1(S25N) complex. This part is a bit longer than the index finger (from 387 to 449), the corresponding sequence is shown. The Figure demonstrates the quality of the electron density (blue mesh). Both of the electron densities are depicted at 1.5 σ. (TIF) [file ppat.1002528.s006.tif]
