# Supplementary material for: Structural Insights into a Unique Legionella pneumophila Effector LidA Recognizing Both GDP and GTP Bound Rab1 in Their Active State
Source: PLoS Pathog. 2012 Mar 1;8(3):e1002528. doi: 10.1371/journal.ppat.1002528 (PMC3295573; doi:10.1371/journal.ppat.1002528)
Supplement: Protocol S1 — Domain mapping by gel filtration. To map the interaction domain of LidA with Rab1, the proteins of LidA(FL) and fragments, Rab1(FL, Q70L) were purified by affinity chromatography with N-terminal His-tag, then mixed protein solutions together at 4°C for 2 h, the protein concentration of Rab1 and LidA was at a molar ratio of 1∶1. Afterwards, the protein mixtures were subject to size exclusion chromatography on a Superdex-200, monitored by UV absorption at 280 nm. The buffer of gel filtration containing 25 mM Tris-HCl (pH 8.0), 100 mM NaCl, and 3 mM DTT. Each aliquots of the peak fraction were subjected to SDS-PAGE which were visualized by Coomassie Brilliant Blue staining. (DOCX) [file ppat.1002528.s007.docx]

**Protocol S1 Domain mapping by gel filtration**

To map the interaction domain of LidA with Rab1, the proteins of LidA(FL) and fragments, Rab1(FL, Q70L) were purified by affinity chromatography with N-terminal His-tag, then mixed protein solutions together at 4^o^C for 2 h, the protein concentration of Rab1 and LidA was at a molar ratio of 1:1. Afterwards, the protein mixtures were subject to size exclusion chromatography on a Superdex-200, monitored by UV absorption at 280 nm. The buffer of gel filtration containing 25 mM Tris-HCl (pH 8.0), 100 mM NaCl, and 3 mM DTT. Each aliquots of the peak fraction were subjected to SDS-PAGE which were visualized by Coomassie Brilliant Blue staining.
