# Supplementary material for: Structural Insights into a Unique Legionella pneumophila Effector LidA Recognizing Both GDP and GTP Bound Rab1 in Their Active State
Source: PLoS Pathog. 2012 Mar 1;8(3):e1002528. doi: 10.1371/journal.ppat.1002528 (PMC3295573; doi:10.1371/journal.ppat.1002528)
Supplement: Protocol S2 — In vitro AMPylation of Rab1a(K62H, 1-176) and purification of AMP-Rab1a(K62H, 1-176). Rab1a(K62H,1-176) protein was AMPylated in the presence of 2.5 molar excess of ATP and 0.01 molar ratio of SidM at room temperature for 4 h, afterwards, the AMPylated Rab1a(K62H,1-176) was purified by gel filtration on a Superdex-200 10/300 column (GE healthcare) at 4°C. Fractions containing AMPylated Rab1a(K62H,1-176) in 20 mM Hepes (pH 8.0), 100 mM NaCl were pooled, concentrated to 1.5 mM, and stored at −80°C. Completeness of AMPylated Rab1a was verified by mass spectrometry as described in mass spectrometry. (DOCX) [file ppat.1002528.s008.docx]

**Protocol S2 In vitro AMPylation of Rab1a(K62H, 1-176) and purification of AMP-Rab1a(K62H, 1-176)**

Rab1a(K62H,1-176) protein was AMPylated in the presence of 2.5 molar excess of ATP and 0.01 molar ratio of SidM at room temperature for 4 h, afterwards, the AMPylated Rab1a(K62H,1-176) was purified by gel filtration on a Superdex-200 10/300 column (GE healthcare) at 4^o^C. Fractions containing AMPylated Rab1a(K62H,1-176) in 20 mM Hepes (pH 8.0), 100 mM NaCl were pooled, concentrated to 1.5 mM, and stored at -80^o^C. Completeness of AMPylated Rab1a was verified by mass spectrometry as described in mass spectrometry.
