# Supplementary material for: Structural Insights into a Unique Legionella pneumophila Effector LidA Recognizing Both GDP and GTP Bound Rab1 in Their Active State
Source: PLoS Pathog. 2012 Mar 1;8(3):e1002528. doi: 10.1371/journal.ppat.1002528 (PMC3295573; doi:10.1371/journal.ppat.1002528)
Supplement: Protocol S3 — Mass spectrometry. Rab1a(K62H,1-176) protein was separated by SDS-PAGE gel, and Coomassie brilliant blue stained. The in-gel digestion of Rab1a(K62H,1-176) for mass spectrometric analysis was performed as published previously [1]. Peptides were dissolved with 0.5% trifluoroacetic acid from digest mixture, and peptide mass analysis was performed using AB4700 MALDI-TOF/TOF mass spectrometer (Applied Biosystems). All data were acquired in the positive ion mode over an m/z range of 500–2000 Da. (DOCX) [file ppat.1002528.s009.docx]

**Protocol S3 Mass spectrometry**

Rab1a(K62H,1-176) protein was separated by SDS-PAGE gel, and Coomassie brilliant blue stained. The in-gel digestion of Rab1a(K62H,1-176) for mass spectrometric analysis was performed as published previously [[1](#_ENREF_1)]. Peptides were dissolved with 0.5% trifluoroacetic acid from digest mixture, and peptide mass analysis was performed using AB4700 MALDI-TOF/TOF mass spectrometer (Applied Biosystems). All data were acquired in the positive ion mode over an m/z range of 500-2000Da.

**Supplementary reference**

1. Shevchenko A, Tomas H, Havlis J, Olsen JV, Mann M (2006) In-gel digestion for mass spectrometric characterization of proteins and proteomes. Nat Protoc 1: 2856-2860.
