# Supplementary material for: Structural Insights into a Unique Legionella pneumophila Effector LidA Recognizing Both GDP and GTP Bound Rab1 in Their Active State
Source: PLoS Pathog. 2012 Mar 1;8(3):e1002528. doi: 10.1371/journal.ppat.1002528 (PMC3295573; doi:10.1371/journal.ppat.1002528)
Supplement: Protocol S4 — Isothermal titration calorimetry. ITC was employed to measure the binding affinities of LidA(188-580) with Rab1a(K62H,1-176) or AMPylated Rab1a(K62H,1-176). All protein samples were purified in a buffer containing 20 mM Hepes (pH 8.0) and 100 mM NaCl. The final concentration of LidA(188-580) were 0.12 mM, Rab1a(K62H,1-176) and AMPylated Rab1a(K62H,1-176) were 1.5 mM. The samples were centrifuged to remove any precipitate before the experiments. Both titrations were performed in the absence of added Mg2+ and ATP. All measurements were carried out at 25°C by using a VP-ITC microcalorimeter 200 (MicroCal). Titrations were carried out by titrating Rab1a(K62H,1-176) or AMPylated Rab1a(K62H,1-176) into LidA(188-580), respectively; The titration Data were analyzed using ORIGIN data analysis software (MicroCalSoftware). (DOCX) [file ppat.1002528.s010.docx]

**Protocol S4 Isothermal titration calorimetry**

af 2.5 molar excess of ATP and as changed into Figure SITC was employed to measure the binding affinities of LidA(188-580) with Rab1a(K62H,1-176) or AMPylated Rab1a(K62H,1-176). All protein samples were purified in a buffer containing 20 mM Hepes (pH 8.0) and 100 mM NaCl. The final concentration of LidA(188-580) were 0.12 mM, Rab1a(K62H,1-176) and AMPylated Rab1a(K62H,1-176) were 1.5 mM. The samples were centrifuged to remove any precipitate before the experiments. Both titrations were performed in the absence of added Mg^2+^ and ATP. All measurements were carried out at 25^o^C by using a VP-ITC microcalorimeter 200 (MicroCal). Titrations were carried out by titrating Rab1a(K62H,1-176) or AMPylated Rab1a(K62H,1-176) into LidA(188-580), respectively; The titration Data were analyzed using ORIGIN data analysis software (MicroCalSoftware).
