# Supplementary material for: Structural Insights into a Unique Legionella pneumophila Effector LidA Recognizing Both GDP and GTP Bound Rab1 in Their Active State
Source: PLoS Pathog. 2012 Mar 1;8(3):e1002528. doi: 10.1371/journal.ppat.1002528 (PMC3295573; doi:10.1371/journal.ppat.1002528)
Supplement: Table S1 — Data collection and structure refinement statistics. (DOCX) [file ppat.1002528.s011.docx]

**Table S1. Data collection and structure refinement statistics.**

|  | LidA(224-559)-Rab1(S25N;1-176) | LidA(188-449)-Rab1(1-191) |
| --- | --- | --- |
| Data Collection | | |
| Space group | *P*2_1_2_1_2_1_ | *C*2 |
| Unit cell dimensions | | |
| a,b,c (Å) | 154.314, 54.673, 61.699 | 128.823, 49.709, 61.702 |
| α,β,γ (^o^) | 90.00, 90.00, 90.00 | 90.00, 99.27, 90.00 |
| Wavelength (Å) | 1.0000 | 1.0000 |
| Resolution (Å) | 50-1.73 | 50-2.2 |
| R_sym_^a^ | 0.072(0.359) | 0.091(0.295) |
| *I/σ (I)* | 23.0(5.4) | 23.9(4.6) |
| Completeness (%, >0 σ ) | 95.99(100)^b^ | 98.52^c^ |
| Redundancy | 3.5(3.4) | 4.2(3.9) |
| Refinement | | |
| Resolution(Å) | 50-1.73 | 50-2.2 |
| No. of reflections (>0 σ) | 53216 | 19986 |
| *R*_work_ ^d^ /*R*_free_ | 19.18/22.79 | 19.22/24.71 |
| No.atoms | | |
| protein | 4267 | 2934 |
| ligand/ion | 28 | 33 |
| water | 389 | 111 |
| R.m.s.d., bond lengths (Å)/angles (^o^) | 0.006/0.960 | 0.009/1.104 |
| Ramachandran plot (%) ^e^ | | |
| Most favoured region | 97.87 | 96.38 |
| Additional allowed region | 2.13 | 3.06 |
| Generously allowed | 0 | 0.56 |
| Disallowed | 0 | 0 |

^a^ Rsym =∑hkl∑i|I(hkl)i -<I(hkl)>|/∑hkl∑I<I(hkl)i> over *i* observations.

^b,c^ Values in parentheses are for reflections in the highest resolution shell.

^d^ *R*_work_ = Σ ||Fo| - |Fc|| / Σ |Fo|, where |Fo| and |Fc| are the observed and calculated structure factor amplitudes, respectively. *R*_free_ for LidA(224-559)-Rab1(S25N;1-176) was calculated with 3.60% of the data. *R*_free_ for LidA(257-449)-Rab1(1-176) was calculated with 5.10% of the data.

^e^ As defined in PROCHECK.
